# Investigation of HCPro-Mediated Ethylene Synthesis Pathway Through RNA-Seq Approaches

**DOI:** 10.3390/v17050602

**Published:** 2025-04-23

**Authors:** Xinpeng Jiang, Lan Dong, Renjing Wan, Changli Zeng, Ting Yang

**Affiliations:** Hubei Engineering Research Center for Protection and Utilization of Special Biological Resources in the Hanjiang River Basin, College of Life Sciences, Jianghan University, Wuhan 430056, China; jiangxinpeng2025@163.com (X.J.);

**Keywords:** ChiVMV, HCPro, ET biosynthesis

## Abstract

Chilli veinal mottle virus (ChiVMV) severely compromises the quality and yield of solanaceous crops. The helper component protease (HCPro) of ChiVMV functions as a multifunctional RNA silencing suppressor that subverts host antiviral defenses through diverse strategies, However, the underlying mechanisms remain mechanistically unresolved. In this study, HCPro-overexpressing (HCPro-OX) and wild-type (WT) plants were inoculated with ChiVMV to monitor the physiological and molecular changes. Transcriptome analysis identified 11,815 differentially expressed genes (DEGs) under viral infection, among which 1115 genes were specifically regulated by HCPro. KEGG enrichment analysis revealed that the DEGs were significantly associated with plant hormone signal transduction pathways, indicating their crucial role in host–virus interactions. Furthermore, functional clustering of HCPro-regulated DEGs specifically identified key components in ethylene biosynthesis pathways. GO analysis of DEGs between virus-inoculated WT and HCPro-OX plants annotated ethylene biosynthesis-related genes *NtACO* and *NtACS*. qPCR validation confirmed that the expression of ethylene biosynthesis-related genes was suppressed by HCPro. Exogenous treatments with the ethylene precursor ACC demonstrated that ethylene suppressed viral accumulation, enhanced POD activity, and reduced the ROS accumulation induced by viral infection. In conclusion, our results demonstrate that HCPro promotes viral infection by suppressing ethylene biosynthesis, which in turn attenuates peroxidase activity, leading to ROS accumulation.

## 1. Introduction

Plants encounter a range of biological stresses during their growth process, such as infections from viruses, bacteria, and fungi. Under these stresses, viral diseases pose a significant threat to plant growth and reproduction [1]. When viruses infect plants, they start making pathogenic proteins, mess up the metabolism of host cells, and increase the accumulation of toxic compounds [2]. Breeding “virus-free” plants or utilizing virus-resistant varieties can effectively mitigate the damage resulting from virus infection. Elucidating the mechanism of virus infection and physiological response of plants to virus infection is an important way to improve plant disease resistance.

ChiVMV is a single-stranded RNA virus with a genome length of approximately 9.7 kb, classified within the family Potyviridae. It is primarily transmitted through aphids, leading to significant agricultural challenges on a continental scale worldwide [3,4,5]. ChiVMV predominantly infects Solanaceae crops, including pepper, tomato, tobacco, and eggplant, serving as its primary natural hosts. In infected capsicum, characteristic symptoms include dark green stripes along leaf veins, leaf shrinkage, and fruit deformation [6]. The virus also induces leaf mottling and necrosis in tobacco plants, ultimately resulting in plant death and posing a severe threat to the tobacco industry [7]. Since its first reported discovery in China in 2003, ChiVMV has proliferated rapidly in intensive agricultural systems, affecting a variety of crops such as pepper, tobacco, and tomato. It has emerged as a significant pathogen threatening agricultural productivity [8]. Therefore, novel strategies are urgently required to mitigate viral infections and enhance plant antiviral resistance.

To facilitate infection, the majority of plant viruses encode viral suppressors of RNA silencing (VSRs), which impede the host’s RNA silencing mechanism [9]. VSRs from different viruses can target distinct stages of the silencing pathway. VSRs commonly inhibit RNA silencing by binding to double-stranded RNA (dsRNA). The key mechanisms involve interacting with HUA enhancer 1 (HEN1), which suppresses its methyltransferase activity. The interaction prevents HEN1 from associating with Argonaute 1 (AGO1) and disrupts the AGO1-dependent homeostasis network [10]. ChiVMV HCPro, as a VSR, exhibits various additional functions. For instance, HCPro has been found to impact heterologous virus infection through salicylic acid and growth hormone pathways. ET signaling genes in P1/HC-Pro plants are significantly enriched in gene-to-gene networks, while endogenous ET also accumulates prominently in P1/HC-Pro plants [11]. ChiVMV can cause severe diseases in plants. However, the mechanism by which HCPro utilizes host factors for modulating viral infection remains unclear.

Viral infections sometimes cause changes in plant hormones. For example, Mal de Río Cuarto virus (MRCV) induces the accumulation of jasmonic acid (JA), brassinolide (BR), abscisic acid (ABA), and indoacetic acid (IAA) in maize and wheat leaf cells, while rice black stripe dwarf virus (RBSDV) seriously interferes with the hormone metabolism of rice and increases the content of ABA and cytokinin (CTK). After virus infection in P1/HC-Pro plants, endogenous ET is highly accumulated in the plants [11]. These results suggest that plant hormone metabolism may be a target of viral attack and that certain plant hormones may play a role in activating resistance mechanisms in plants. Previous studies have shown that the interaction between turnip mosaic virus HCPro and SA-binding protein 3 disrupts the SA pathway, thereby suppressing the host’s defense response and promoting viral infection [12]. Additionally, SA inhibits the replication of tomato bushy stunt virus by competitively binding to cytosolic glyceraldehyde-3-phosphate dehydrogenase (GAPDH) [13]. Another study revealed that ABA suppresses the transcription of a basic β-1,3-glucanase, thus forming a physical barrier that restricts viral movement through plasmodesmata [14]. Plant hormones such as SA, JA, and ET play a vital role in a mature defense system against pathogen attack. ET plays a dual role in plant physiology, contributing to both growth and defense mechanisms, which makes it particularly noteworthy. ET is synthesized by its precursor 1-aminocyclopropane-1-carboxylic acid (ACC), which is regulated by 1-aminocyclopropane-1-carboxylic acid oxidase (*ACO*). Studies have shown that rice dwarf virus (RDV) can stimulate the production of ET by enhancing the activity of S-adenosine-L-methionine synthetase (*SAMS*), thereby increasing the susceptibility of rice plants to RDV infection [15]. JA and ET signaling are essential for the systemic resistance of tobacco against tobacco mosaic virus (TMV) [16]. These findings suggest that ET may play an active regulatory role in resisting viral attack, but its exact role and mechanism require further study.

In this study, plants overexpressing ChiVMV HCPro were investigated to uncover HCPro-mediated regulation of ET biosynthetic pathways. The aim of this study was to understand the mechanism of virus regulation of host plant factors to promote virus infection and explore the role and potential mechanism of ET in plant disease resistance.

## 2. Materials and Methods

### 2.1. Plant Materials

Wild-type (WT) and HCPro-overexpressing (HCPro-OX) plants of *N. tabacum* were utilized in this experiment, and the HCPro-OX plants were obtained in our previous study [17]. The seeds were sown in a soil mixture consisting of coconut soil, nutrient soil, and vermiculite at a ratio of 3:2:1. The plants were cultivated in a greenhouse at 23 °C ± 2 °C with a light–dark cycle of 15 h and 9 h, respectively. The seedlings with essentially the same growth trend were chosen as the research objects in the research. Five-week-old seedlings (WT and HCPro-OX) were inoculated with ChiVMV on the third and fourth leaves, with 10 μL of virus inoculated on each leaf, and the control check (CK) was performed by adding phosphate-buffered saline (PBS) without the virus to the leaves.

### 2.2. cDNA Library Construction and RNA Sequencing

Leaves from CK and virus-inoculated plants (V) were collected for RNA-seq analysis, with each group consisting of four biological replicates. Total RNA was extracted from *N. tabacum* leaves, and then the library was sequenced by Biomarker Technologies Co., Ltd. (Beijing, China) on Illumina Nova Seq platform to generate 150 bp double-terminal sequence. The raw readings were further processed using the bioinformatics analysis platform BMK Cloud (www.biocloud.net, accessed on 10 August 2023). Clean data were obtained by removing sequences that contain joints, sequences that contain poly-N, and sequences of low quality from the original data. The Q20, Q30, GC content, and sequence repetition level were calculated at the same time.

All downstream analyses were based on high-quality clean data. Valid data were matched to a reference genome sequence. Based on the reference genome, only sequences that were a perfect match or had a small number of mismatches were further analyzed and annotated. The Hisat2 tool software (version 2.0.4) was used to align the sequences with the reference genome [18]. Gene functions are annotated by sequence alignment based on the following databases: NCBI non-redundant protein sequences (Nr); Pfam (protein family); KOG/COG (clusters of orthologous groups of proteins); Swiss-Prot (a manually annotated and reviewed protein sequence database); KO (KEGG ortholog database); GO (gene ontology).

### 2.3. Differential Gene Expression Analysis

The “DESeq” R software package (version 1.18.0) was utilized for conducting differential expression analysis of the following pairwise comparisons: WT-CK vs. WT-V, HCPro-CK vs. HCPro-V, WT-CK vs. HCPro-CK, WT-V vs. HCPro-V [19]. Statistical procedures were employed to determine differential expression in digital gene expression data using a model that incorporates the negative binomial distribution. The resulting *p*-values were adjusted using Benjamini and Hochberg’s method to control the false discovery rate, the DEGs were selected based on thresholds of |log2 fold change| ≥ 1 with *p*-value ≤ 0.05.

### 2.4. Expression-Based Heatmaps and Principal Component Analysis (PCA)

Generating heatmaps of DEG expressing with the R tool (version 4.4.1). The R software package ggplot2 (version 3.5.1)was used for principal component (PCA) and correlation analysis [20]. PCA was performed on all transcripts obtained from individual replicates using FPKM values.

### 2.5. GO Enrichment Analysis and KEGG Pathway Enrichment Analysis

GO enrichment analysis was performed using the top GO program (version 2.48.0) [21]. Using the Blast 2 GO program, all DEGs were annotated in the GO database. DEGs with a GO term were used to calculate both the gene list and gene number for each term. Then, the *p*-value was calculated with the hypergeometric distribution method to identify the GO term for significant enrichment of DEGs compared with the whole genome background, to determine the main biological functions of the DEGs.

KEGG was performed to explore the pathways associated with DEGs [22]. The significantly enriched pathways were screened using Fisher’s exact test based on hypergeometric distribution to identify the biological functions of genes. An enriched pathway *p*-value ≤ 0.05 was deemed statistically significant.

### 2.6. External Application of Hormones

ACC was dissolved in ethanol, and pyrazinamide (PZA) was dissolved in dimethyl sulfoxide. Then, the stock solutions were diluted to their final concentrations: ACC (200 μM/L), PZA (20 mg/mL). Twenty-four hours before virus inoculation, *N. tabacum* plants were sprayed with the above-mentioned prepared solutions. At least ten plants were used for each treatment. Distilled water was used as a control treatment. The experimental results were obtained by at least three independent biological repetitions.

### 2.7. Staining with Nitro Blue Tetrazolium and 3,3′-Diaminobenzidine

Superoxide (O^2−^) and hydrogen peroxide (H_2_O_2_) were visually detected with nitro blue tetrazolium (NBT) and 3,3′-diaminobenzidine (DAB), as described previously [23]. Fresh leaves were cut from the base of the petiole and then soaked in NBT solution for 4 h or DAB solution for 6 h. The leaves were decolorized with 95% ethanol in a boiling water bath.

### 2.8. Assays for Hydrogen Peroxide

Lipid peroxidation was estimated with a malondialdehyde (MDA) detection kit (Nanjing Jiancheng) by measuring the absorbance at 532 nm. The H_2_O_2_ content was quantified using a colorimetric assay following the manufacturer’s instructions for the H_2_O_2_ assay kit (Nanjing Jiancheng, Nanjing, China). Fresh tobacco leaves (2.0 g) were washed to remove surface impurities and then homogenized with 2 mL of 25 mM phosphate buffer (pH = 6.8). The resulting homogenate was centrifuged at 8000 rpm and 4 °C for 10 min, and 100 μL of the supernatant was used for further analysis by reacting with the substrate. The absorbance value was measured at 405 nm using a microplate reader.

### 2.9. Total RNA Extraction, Reverse-Transcription PCR, and Quantitative PCR Analysis

Tobacco leaves were placed in a mortar and ground with liquid nitrogen until the tissue was completely powdered. Subsequently, 0.1 g of the powdered sample was weighed and extracted for total RNA using the Eastep Super Total RNA Extraction Kit following the instruction manual. cDNA for PCR was synthesized using M-MLV reverse transcriptase I. RT-qPCR was conducted using the AceQ Universal SYBR qPCR Master Mix kit (Vazyme Biotech Co., Ltd., Nanjing, China), as per the manufacturer’s protocol. Quantitative data analysis was performed using the 2^−ΔΔCT^ method, while extension factor 1α (*EF1α*) gene served as an internal control for transcriptional level standardization. The primers used for the RT-qPCR assays are listed in Table S1.

### 2.10. Statistical Analyses

Means were calculated from at least three biological replicates. For statistical comparisons between different treatments, one-way analysis of variance followed by Duncan’s multiple range test was employed [24]. Differences were considered statistically significant at *p* < 0.05. Figures were generated using GraphPad Prism 9.

## 3. Results

### 3.1. Transcriptome Sequencing of N. tabacum Leaves Infected with ChiVMV

**Figure 1:**
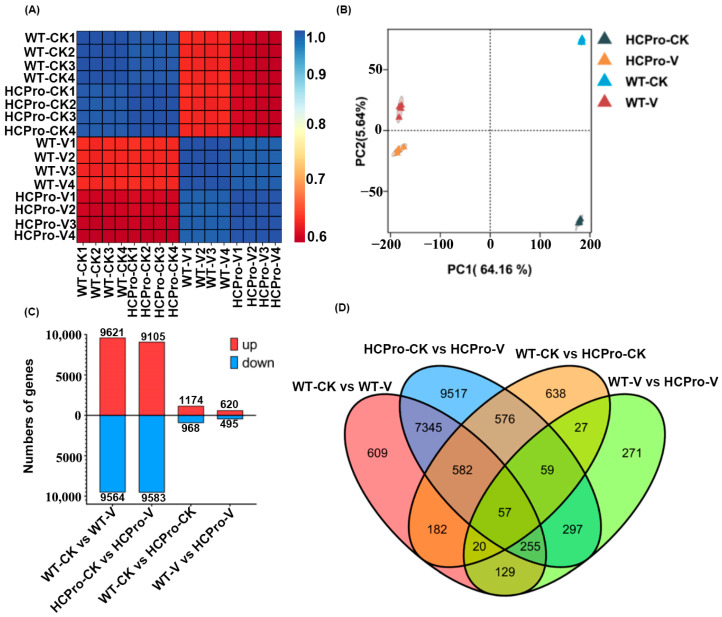
Exploratory data analysis and RNA-seq analysis. (**A**) Hierarchical clustering of samples based on log2 transformed gene counts showing a correlation between samples. The redder the color, the smaller the distance between the samples. (**B**) Principal component analysis plot of normalized and variance stabilized counts from DESeq2. Samples with similar expressions are grouped closely. (**C**) Differentially expressed genes in the groups “WT-CK vs. WT-V”, “WT-CK vs. HCPro-CK”, “HCPro-CK vs. HCPro-V”, and “WT-V vs. HCPro-V”. (**D**) Venn diagram representing the differentially expressed genes overlapping between four groups.

The leaves of WT plants and HCPro-OX plants were collected for transcriptome sequencing, and 102.10 Gb of clean reads was obtained. The clean reading of each samples reached 5.98 Gb, and the percentage of Q30 bases was 93.58% or above (Table S2). The sequencing results exhibited high correlation (r^2^ > 0.996) and reproducibility (Figure 1A). The results of the principal component analysis (PCA) showed significant differences in PCA values between different samples, with principal component 1 (PC1) and principal component 2 (PC2) explaining 64.16% and 5.64% of the total variance, respectively; the results indicated the extent of variation between treatments (Figure 1B). These results indicate that there are significant differences in transcription levels between WT plants and HCPro-OX plants after virus infection. Compared to CK, 19,185 and 18,688 DEGs were detected in virus-infected WT plants and virus-infected HCPro-OX plants, respectively. In the group “WT-CK vs. HCPro-CK”, 2141 DEGs were detected between WT plants and HCPro-OX plants. In addition, a comparative analysis of WT-V and HCPro-V plants identified 1115 DEGs (Figure 1C). Additionally, 57 DEGs were commonly identified among all comparisons (Figure 1D).

### 3.2. The Expression of ChiVMV HCPro Promotes Virus Infection

**Figure 2:**
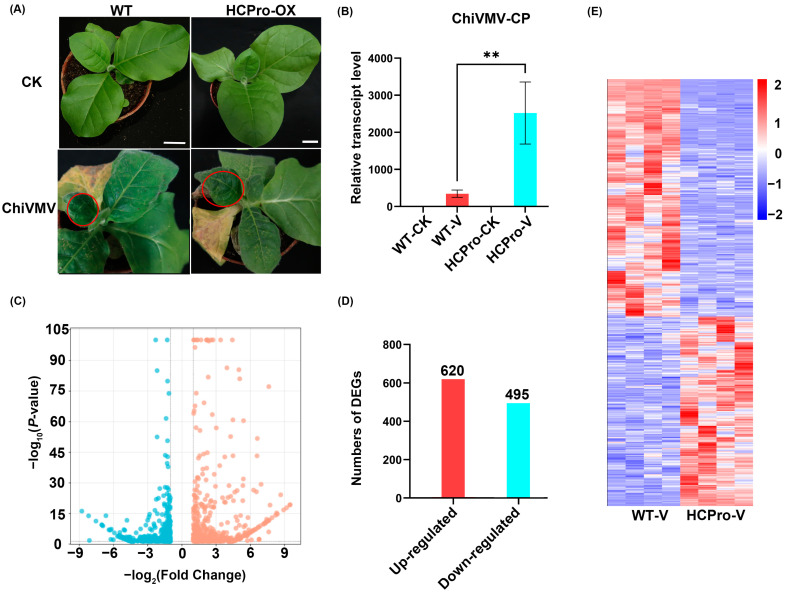
The response of HCPro-OX plants to virus infection. (**A**) Symptoms of WT and ChiVMV HCPro leaves’ systemic infection with ChiVMV. Red circles highlighting symptoms of plant disease. (**B**) RT-qPCR detection of the relative RNA level of ChiVMV-CP in leaves of systemically infected plants. ** *p*-value < 0.01. (**C**) Differentially expressed genes in the group “WT-V vs. HCPro-V”. (**D**) Number of upregulated and downregulated genes after virus inoculation. (**E**) Heat maps of DEGs of WT plants and HCPro-OX plants after inoculation with ChiVMV.

To investigate the effect of HCPro overexpression on ChiVMV infection, we first observed symptoms and measured the accumulation of the virus in the leaves of infected plants. After inoculation with the virus, the leaves showed signs of mottling and necrosis, and HCPro-OX plants showed more severe symptoms than WT plants (Figure 2A). The results of RT-qPCR showed that the relative RNA level of the coat protein of ChiVMV (ChiVMV-CP) in WT plants was lower than that in HCPro-OX plants (Figure 2B). These results indicate that overexpression of ChiVMV HCPro in *N. tabacum* can promote virus infection. The |log2 fold change| ≥ 1 with *p*-value ≤ 0.05 was defined as DEGs in the group “WT-V vs. HCPro-V”. It was found that 620 and 495 genes were up- and downregulated (Figure 2C,D). An overview of the transcriptomic profiles and hierarchical clustering analysis revealed a substantial number of DEGs between the WT plants and HCPro-OX plants following viral inoculation (Figure 2E).

### 3.3. Functional Annotation of DEGs in N. tabacum Leaves Infected with ChiVMV

**Figure 3:**
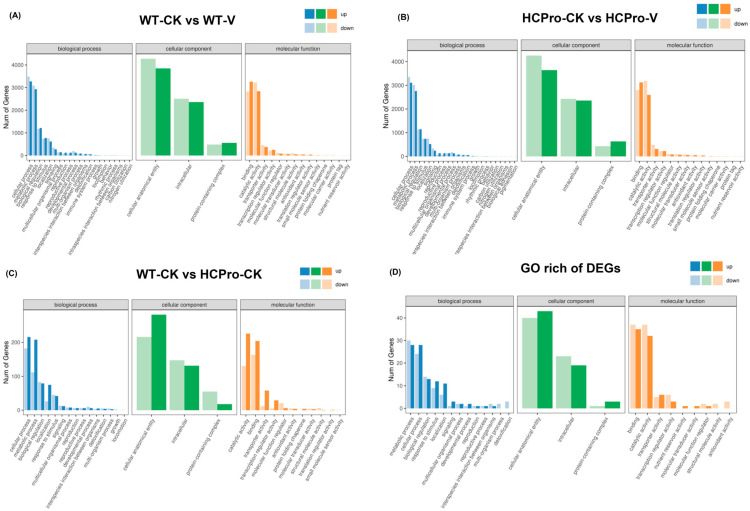
GO analysis of DEGs. (**A**) Enriched GO terms DEGs in “WT-CK vs. WT-V”. (**B**) Enriched GO terms DEGs in “HCPro-CK vs. HCPro-V”. (**C**) Enriched GO terms DEGs in “WT-CK vs. HCPro-CK”. (**D**) Enriched GO terms of the same DEGs in “WT-CK vs. WT-V”, “HCPro-CK vs. HCPro-V” and “WT-CK vs. HCPro-CK”.

To better explain the biological functions of DEGs, GO enrichment analysis was carried out for the plants, the up- and downregulated DEGs were annotated with GO terms and assigned to biological process, cellular component, and molecular function (Figure 3).

The results showed that a total of 14 biological processes, 3 cellular components, and 10 molecular functions were significantly enriched in the “WT-CK vs. WT-V” group, including “metabolic process” (GO:0008152) and “response to stimulus” (GO:0050896) (Figure 3A). The “HCPro-CK vs. HCPro-V” group revealed 15 biological processes, 3 cell components, and 10 molecular functions including “response to stimulus” (GO:0050896), “metabolic process” (GO:0008152), and “catalytic activity” (GO:0003824) (Figure 3B). “WT-CK vs. HCPro-CK” group revealed 13 biological processes, 3 cell components, and 10 molecular functions, among which “metabolic process” (GO:0008152) and “signaling” (GO:0023052) were significantly enriched (Figure 3C).

The analysis revealed significant functional enrichment across the groups “WT-CK vs. WT-V”, “HCPro-CK vs. HCPro-V”, and “WT-CK vs. HCPro-CK”, with 11 biological processes, 3 cellular components, and 7 molecular functions consistently associated with the shared DEGs, including “response to stimulus” (GO:0050896), “metabolic process” (GO:0008152), “catalytic activity” (GO:0003824), and “antioxidant activity” (GO:0016209) (Figure 3D). Notably, the analysis of the same DEGs among the above three groups revealed that HCPro likely suppressed the expression of genes associated with the “antioxidant activity” pathway (GO:0016209) during viral infection, thereby disrupting cellular redox homeostasis mechanisms.

**Figure 4:**
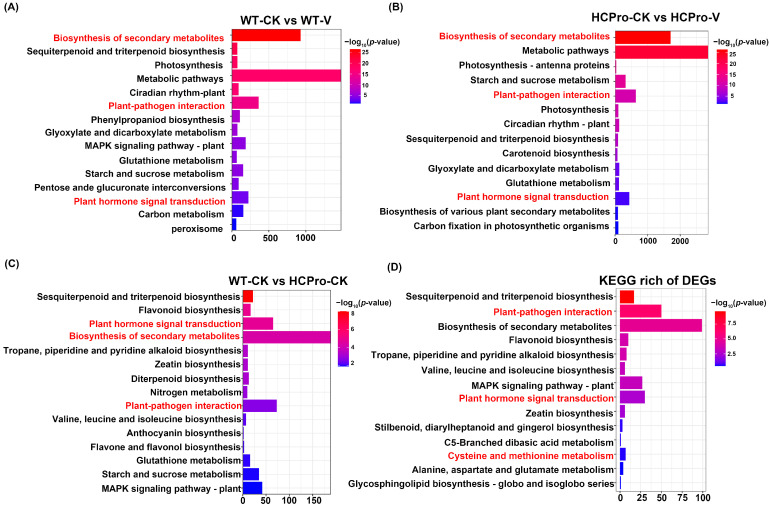
KEGG enrichment pathway is based on the differentially expressed genes in groups (**A**) WT-CK vs. WT-V, (**B**) HCPro-CK vs. HCPro-V, and (**C**) WT-CK vs. HCPro-CK. (**D**) Common differentially expressed genes of “WT-CK vs. WT-V”, “HCPro-CK vs. HCPro-V”, and “WT-CK vs. HCPro-CK”.

To further investigate the role of HCPro in modulating plant disease resistance, KEGG enrichment analysis was performed on the DEGs identified across above three comparison groups: “WT-CK vs. WT-V”, “HCPro-CK vs. HCPro-V”, and “WT-CK vs. HCPro-CK”. The results revealed that in the “WT-CK vs. WT-V” group, the DEGs were significantly enriched in pathways such as “plant hormone signal transduction” (ko04075), “plant-pathogen interaction” (ko04626), “biosynthesis of secondary metabolites” (ko01110), “MAPK signaling pathway—plant” (ko04016) and others (Figure 4A). In the “HCPro-CK vs. HCPro-V” group, the DEGs were primarily enriched in “plant hormone signal transduction” (ko04075), “metabolic pathways” (ko01100), and “plant-pathogen interaction” (ko04626) (Figure 4B). In addition, for the “WT-CK vs. HCPro-CK” group, the DEGs showed enrichment in “MAPK signaling pathway—plant” (ko04016), “plant hormone signal transduction” (ko04075), and “plant-pathogen interaction” (ko04626) (Figure 4C). The results showed that these DEGs are enriched in “plant hormone signal transduction” (ko04075) and “plant-pathogen interaction” (ko04626).

Enrichment analysis of the DEGs across the above three groups was conducted to further explore their functional roles, revealing significant enrichment in the “cysteine and methionine metabolic” (ko00270), within this pathway, DEGs exhibited pronounced enrichment in ET biosynthesis-related processes (Figure 4D). These results indicated that HCPro likely enhances viral infection by interfering with plant hormone pathways and interfering the expression of genes associated with ET biosynthesis.

### 3.4. HCPro Modulates ET Biosynthesis in Plants During ChiVMV Infection

**Figure 5:**
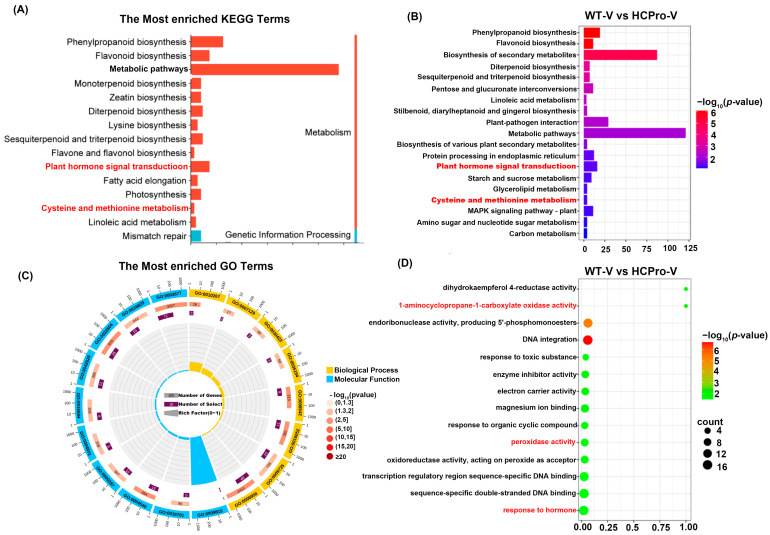
DEGs in the group WT-V vs. HCPro-V. (**A**) The KEGG pathway of the DEGs in the group “WT-V vs. HCPro-V”. (**B**) KEGG enrichment pathway is based on the differentially expressed genes in “WT-V vs. HCPro-V”. (**C**) The go pathway of the DEGs in the group “WT-V vs. HCPro-V”. (**D**) GO enrichment is based on the differentially expressed genes in “WT-V vs. HCPro-V”.

KEGG enrichment analysis was performed on the DEGs identified in the “WT-V vs. HCPro-V” group. The analysis revealed significant enrichment across three functional categories: 14 biological processes, 3 cellular components, and 10 molecular functions, including “plant hormone signal transduction” (ko04075) and “cysteine and methionine metabolic” (ko00270). Notably, integrated analysis with the above analysis demonstrated that HCPro modulates phytohormone signaling pathways and peroxidase activity. Specifically, DEGs in this comparison were enriched in phytohormone regulatory networks and the ET biosynthesis pathway (Figure 5A,B).

GO enrichment analysis was performed on the “WT-V vs. HCPro-V” group. The results revealed a significant enrichment of DEGs in three critical functional categories: “1-aminocyclopropane-1-carboxylate oxidase activity” (GO:0009815), “peroxidase activity” (GO:0004601), and “response to hormones” (GO:0009725). These findings aligned with and further validated our above hypothesis that HCPro interferes with ethylene-mediated defense mechanisms and redox homeostasis (Figure 5C,D). In particular, the genes related to ethylene biosynthesis were inhibited, and the activity of peroxidase was also suppressed, collectively highlighting the role of HCPro in interfering with plant immune responses.

**Figure 6:**
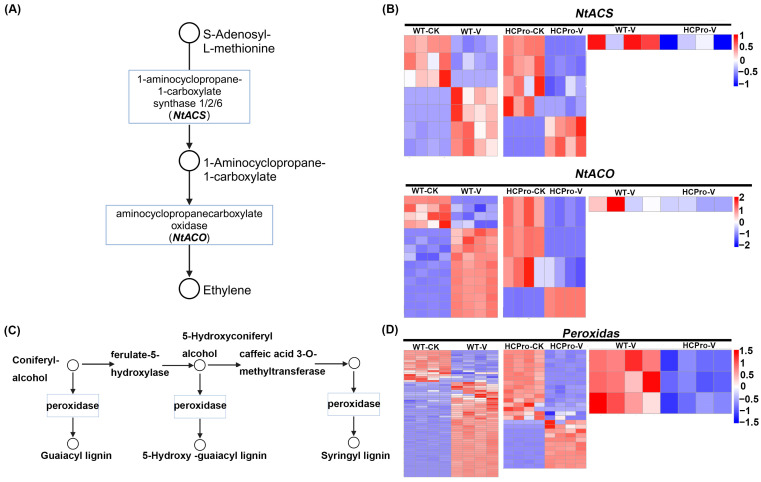
The DEGs of ethylene biosynthesis pathway and peroxidase in the group “WT-V vs. HCPro-V”. (**A**) Differentially expressed genes in ethylene biosynthesis pathway. (**B**) Heat maps of DEGs in ethylene biosynthesis pathway. (**C**) Differentially expressed genes of peroxidase in the KEGG pathway. (**D**) Heat maps of peroxidase.

A targeted analysis of “cysteine and methionine metabolism” (ko00270), which is linked to ET biosynthesis, revealed differential regulation of key ET biosynthesis across the groups “WT-CK vs. WT-V”, “HCPro-CK vs. HCPro-V”, and “WT-V vs. HCPro-V”, particularly *NtACS* and *NtACO* (Figure 6A).

Comparative transcriptomic profiling of WT and HCPro-OX plants before and after viral inoculation was affected to varying degrees in ET biosynthesis-related genes (Figure 6A,B). While all plants exhibited both up- and downregulation of these genes after viral inoculation relative to control plants, HCPro-OX plants showed preferential downregulation of the gene expression relative to WT plants (Figure 6B). Notably, a direct comparison between virus-infected WT plants and HCPro-OX plants revealed that the latter exhibited significantly stronger suppression of *NtACS* and *NtACO*, demonstrating that HCPro can inhibit the biosynthesis of ET, thereby promoting the infection of the virus.

To further characterize genes associated with peroxidase activity, transcriptomic profiling revealed a moderate upregulation of peroxidase-related genes (Figure 6C). In WT plants, viral inoculation triggered moderate upregulation of these genes compared to CK, suggesting that infection activates peroxidase-mediated oxidative stress responses. Strikingly, while peroxidase genes in HCPro-OX plants were dynamically modulated after virus inoculation, the expression was markedly suppressed compared to WT plants (Figure 6D). It implied that HCPro can disrupt redox homeostasis mechanism to promote virus infection.

### 3.5. Validation of Differentially Expressed Genes in HCPro-Regulated Pathways

**Figure 7:**
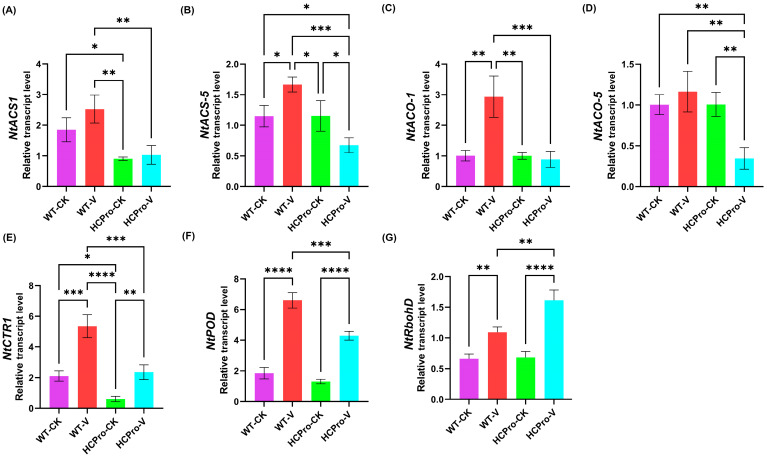
The qRT-PCR-based validation of the gene expressions in the plants. (**A**) 1-aminocyclopropane-1-carboxylic acid synthase 1 (*NtACS-1*). (**B**) 1-aminocyclopropane-1-carboxylic acid synthase 1 (*NtACS-5*). (**C**) 1-aminocyclopropane-1-carboxylic acid oxidase 1 (*NtACO-1*). (**D**) 1-aminocyclopropane-1-carboxylic acid oxidase 5 (*NtACO-5*). (**E**) Constitutive triple response 1 (*NtCTR1*). (**F**) Peroxidase (*NtPOD*). (**G**) Respiratory burst oxidase homologue (*NtRboh*). * *p*-value < 0.05, ** *p*-value < 0.01, *** *p*-value < 0.001, **** *p*-value < 0.0001.

RT-qPCR was performed to measure the expression levels of five key genes in the ET pathway and two key genes in the peroxidase pathway, thereby validating the reliability of the RNA-seq data. As shown in Figure 7, the expression patterns detected by RT-qPCR closely matched those identified through transcriptomics, confirming the robustness of the RNA-seq results. The results showed that the expression of *NtACO* and *NtACS* were significantly lower in HCPro-OX plants than in WT plants after virus inoculation (Figure 7A–D), suggesting that HCPro suppresses ET biosynthesis in plants. The expression of *NtPOD* was suppressed in HCPro-OX plants, whereas the *NtRbohD* associated with ROS burst was exhibited upregulation (Figure 7F,G). These findings suggested that HCPro disrupts redox homeostasis in plants by modulating the expression of *NtPOD*, thereby promoting viral infection.

### 3.6. ET Plays a Positive Role in the Response of N. tabacum to ChiVMV

**Figure 8:**
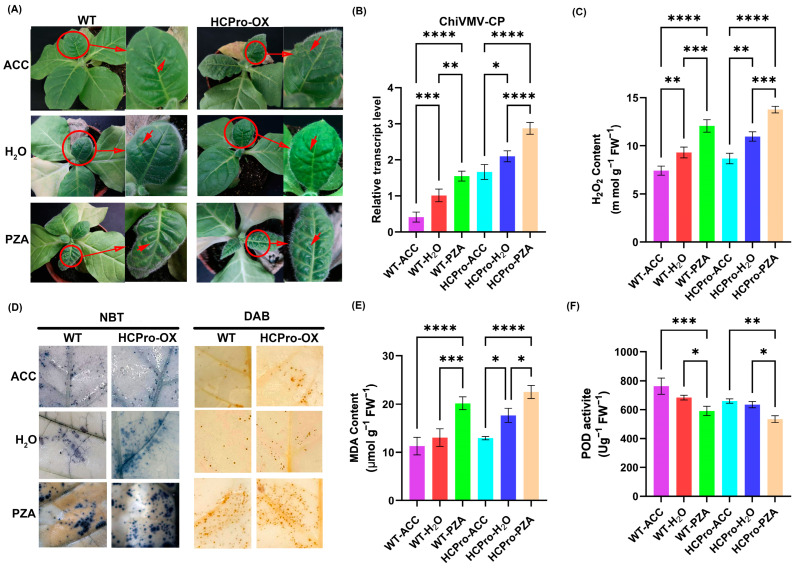
Validation of external hormone application. (**A**) Symptoms of WT and HCPro-OX plant leaves after inoculation with the virus for external application of ACC, water, and PZA. Red circles highlight the diseased leaves. A long arrow points to the magnified view of the diseased leaf, while short arrows draw attention to specific plant disease symptoms (**B**) RT-qPCR detection of the relative RNA level of ChiVMV-CP in leaves of systemically infected plants after external treatment. (**C**) H_2_O_2_ content after inoculation with the virus and external treatment. (**D**) Nitroblue tetrazole (NBT) stained and 3,3′-diaminobenzidine (DAB) stained after inoculation with the virus and external treatment. (**E**) MDA content after virus inoculation and external treatment. (**F**) POD activity after virus inoculation and external treatment. * *p*-value < 0.05, ** *p*-value < 0.01, *** *p*-value < 0.001, **** *p*-value < 0.0001.

To further explore the role of ET in promoting virus infection, virus-infected WT plants and HCPro-OX plants were treated with ACC and PZA. The results showed that both WT and HCPro-OX plants treated with ACC exhibited significantly milder symptoms compared to water-sprayed plants, whereas PZA-treated plants displayed exacerbated symptom severity (Figure 8A). RT-PCR analysis revealed that HCPro-OX plants consistently accumulated higher ChiVMV-CP levels than WT plants with the same treatments, and it confirmed the role of HCPro in promoting viral infection. In virus-inoculated WT plants, PZA treatment increased the accumulation of ChiVMV compared to water-treatment, the virus level was the lowest in the plants treated by ACC. Similarly, HCPro-OX plants displayed enhanced viral accumulation under PZA treatment compared to water plants, whereas ACC treatment achieved maximum viral suppression (Figure 8B). The findings indicated that ET enhances plant antiviral activity and that HCPro likely facilitates infection by interfering with ET-mediated defense mechanisms.

ROS also play a crucial role in plant defense mechanisms. As shown in Figure 8C, viral infection induced H_2_O_2_ accumulation, and HCPro-OX plants exhibited a significantly higher H_2_O_2_ content than WT plants with identical treatment, indicated that HCPro enhanced ROS accumulation in virus-infected plants. In WT plants, PZA-treated plants showed elevated H_2_O_2_ levels compared to water-treated plants, while ACC-treated WT plants displayed the lowest H_2_O_2_ content. Similarly, in HCPro-OX plants, PZA treatment increased H_2_O_2_ accumulation relative to water-treated plants, whereas ACC-treated HCPro-OX plants showed minimal H_2_O_2_ levels. These findings confirmed that ET promotes ROS scavenging in plants.

Superoxide and H_2_O_2_ accumulation in systemically infected leaves were visualized using NBT and DAB staining, respectively. Consistently, HCPro-OX plants exhibited darker staining intensities than WT plants within the same treatment, reflecting of elevated ROS accumulation. In WT plants, PZA treatment intensified staining compared to water-treated plants, while ACC-treated plants showed the faintest staining. Analogously, HCPro-OX plants treated with PZA displayed deeper staining than water-treated plants, with ACC-treated plants exhibiting minimal staining. These results further corroborated that ET enhances ROS clearance (Figure 8D). All staining patterns aligned quantitatively with H_2_O_2_ measurement data.

After virus inoculation, HCPro-OX plants exhibited significantly higher MDA levels than WT plants within the same treatments. In WT plants, PZA-treated plants showed elevated MDA content compared to water-treated plants, while ACC-treated WT plants displayed the lowest MDA accumulation. Similarly, HCPro-OX plants treated with PZA demonstrated increased MDA levels relative to water-treated plants, with ACC-treated HCPro-OX plants exhibiting minimal MDA content (Figure 8E). An identical trend was observed for peroxidase activity (Figure 8F). These findings collectively demonstrated that HCPro enhances oxidative damage in plants by suppressing POD activity, while ET alleviates this stress by enhancing the activity of peroxidase.

## 4. Discussion

As a major pathogen infecting Solanaceae crops, ChiVMV infects tobacco plants and will induce patchy yellow symptoms, which severely impairs plant growth. Investigating the response of tobacco to viral infection can provide valuable insights into developing strategies to mitigate yield losses caused by viral diseases. During the process of plant–virus interaction, plant viruses evolve mechanisms to counteract host defenses through sequence changes such as mutation, recombination, natural selection, gene drift, or migration. To successfully invade the host and evade these defense mechanisms, pathogens develop RNA-silencing viral suppressors [25,26]. The HCPro protein is the first identified RNA-silencing suppressor in the potyvirus family. It is a multifunctional protein that inhibits RNA silencing and plays roles in viral virulence, movement, and transmission; its coding sequence exhibits high variability and can inhibit RNA silencing to counteract the plant’s defense response [27,28].

Previous studies have demonstrated that ChiVMV HCPro interacts with catalase isoform CAT1, effectively suppressing its enzymatic activity [17]. This specific interference leads to the accumulation of hydrogen peroxide, which facilitates viral pathogenesis by creating a redox environment favorable for viral replication. However, different inhibitory proteins may have distinct roles in various plant–virus interaction systems [29,30,31]. The transfer of VSRs from different viruses into *benthamiana* or *N. tabacum* resulted in diverse phenotypes including strongly curled leaves, flowers with petioles absent, or malformed flowers [32]. In this study, the overexpression of ChiVMV HCPro in *N. tabacum* had no significant negative effects on the growth and development of *N. tabacum* plants. In the early stage of growth, the petiole was extended, the leaves were slightly curled, and the internode was slightly longer in the later stage. Thus, it can be inferred that phenotypic differences between WT plants and HCPro-OX plants are due to HCPro rather than ChiVMV itself.

Through transcriptome sequencing, we obtained comprehensive information regarding the alterations in global gene expression between WT and HCPro-OX plants upon ChiVMV infection. By conducting a thorough analysis of these RNA-seq data, we successfully identified differentially expressed genes associated with distinct regulatory pathways. Our hypothesis suggests that differential genes involved in ethylene biosynthesis play a pivotal role in tobacco’s response to ChiVMV infection. For instance, GO and KEGG pathway analyses revealed the induced expression changes in multiple stress response genes, plant–pathogen interaction-related genes, and ethylene biosynthesis-associated genes following ChiVMV infection. Additionally, the findings indicate the inhibition of peroxidase-associated genes.

The presence of viral infections has been observed to induce alterations in plant hormone levels across a diverse range of crops. Studies have shown the effect of ChiVMV HCPro expression on the resistance of tobacco plants to cucumber mosaic virus and tobacco mosaic virus, revealing that HCPro can regulate heterologous virus infection through salicylic acid and auxin signaling pathways [33]. In addition, the study found that an ET-induced transcription factor *RAV2* (Related to *ABI3/VP12*) in Arabidopsis Thaliana is essential for the anti-silencing activity of turnip mosaic virus HCPro [34]. Among many hormones, ET, as a key plant hormone, plays a crucial role in promoting fruit ripening, leaf aging, root hair formation, and breaking seed and bud dormancy [35]. The ET response element AP2/ERF plays an important role in the plant response to stress [36,37]. In addition, ET has been shown to actively participate in abiotic and biological stress responses in plants. Studies have shown that the Arabidopsis mutants 1-aminocyclopropane-1-carboxylate synthase 6 (*ACS6*), ET responsive factor 104 (*ERF104*), and ET insensitivity 2 (*EIN2*) are resistant to TMV-cg infection [38]. In addition, ACC application promoted the accumulation of TMV in treated plants, the study also showed that *ACS6* and *ERF104* were significantly upregulated in *WRKY8* mutants, while emphasizing that *WRKY8* negatively regulates these genes by binding to the W-box cluster within its promoter [38].

Some studies have shown that rice dwarf virus induces ET production by stimulating S-adenosine-L-methionine synthase (SAMS), a key component of the ET biosynthesis pathway. This results in the increased susceptibility of rice to RDV [15]. *NtACO* acts as an oxidase to catalyze the precursor ACC to synthesize ET. Studies have shown that various stressors such as virus infection, temperature fluctuation, and drought can increase the endogenous ET level of plants [39]. These studies found that ET plays an important role in the resistance to pathogen infection. In this study, it was found that the expression of *NtACS* and *NtACO* related to ET biosynthesis was inhibited in HCPro-OX plants after inoculation with ChiVMV compared with WT plants, which affected the accumulation of ET in HCPro-OX plants. This suggests that ET may play an important role in antiviral activity. This study provides new insights into the way ChiVMV HCPro promotes viral infection. In this study, we applied ACC and PZA to plants inoculated with the virus, further confirming that ET plays a positive role in enhancing antiviral activity. The data in this study suggest that ET may induce a resistant response to ChiVMV, further illustrating the role of this plant hormone in antiviral infection.

In response to biological and abiotic stresses, plants produce ROS within their cells to induce a defense response. However, excessive ROS accumulation can impair cell structure and physiological function. Antioxidant enzymes such as CAT, POD, and SOD play a crucial role in the removal of ROS. Plant hormones are involved in enhancing resistance to biological and abiotic stresses by stimulating the activity of antioxidant enzymes. ROS controls many different processes in plants, and ROS bursts strengthen cell walls by cross-linking glycoproteins and activating defense signaling components, thus constituting an early response to pathogen attack [40]. Studies have shown that ET can increase peroxidase activity in plants under stress. ET pretreatment can improve plant resistance to hydrogen peroxide, which is related to ET promoting the activity of antioxidant enzymes [41]. Overexpression of the ET responsive factor (*ERF38*) reduced membrane lipid peroxidation, ROS accumulation, and proline and soluble protein accumulation and increased POD and SOD activities [42]. ET can induce the increase of peroxidase activity in barley [43]. Exogenous ET can induce ascorbate peroxidase activity in soybean cells. In this study, ROS accumulation and changes in antioxidant enzyme activity were consistent with the severity of symptoms of viral infection in WT and HCPro-OX plants, respectively. The results showed that ChiVMV infection may lead to ROS accumulation, which may lead to plant cell damage, while ET can stimulate peroxidase activity, thereby reducing ROS levels, and HCPro inhibits ET biosynthesis, resulting in an increase in ROS levels.

## 5. Conclusions

This study analyzed the response of *N. tabacum* to ChiVMV infection using RNA sequencing. The results suggest that ethylene may play an important role in the defense response to ChiVMV infection. In addition, the expression of the ET biosynthesis-related genes *NtACO* and *NtACS* in HCPro-OX plants was downregulated after ChiVMV infection, which inhibited ET biosynthesis and promoted virus infection. Further studies showed that plant peroxidase activity was inhibited in HCPro-OX plants after ChiVMV infection. Previous studies have shown that when plants are stressed, ET can boost peroxidase activity, removing excess reactive oxygen species to achieve the effect of stress resistance. The results showed that the symptoms of the plants treated with ACC and PZA were obviously alleviated. The symptoms of PZA-treated plants were more severe than those sprayed with water, and the H_2_O_2_ content of ACC-treated plants was lower than that of PZA-treated plants. These results suggest that HCPro can inhibit the synthesis of ET, thereby affecting the activity of peroxidase, and thereby inhibiting the clearance of ROS in plants, thus achieving the role of promoting viral infection.

## Data Availability

The raw data supporting the conclusions of this article will be made available by the authors on request.

## Supplementary Materials

The following supporting information can be downloaded at: https://www.mdpi.com/article/10.3390/v17050602/s1, Table S1: Primers used for quantitative real-time PCR analysis. Table S2: Basic summary of RNA-sequencing results.
