# Supplementary material for: Investigation of HCPro-Mediated Ethylene Synthesis Pathway Through RNA-Seq Approaches
Source: Viruses. 2025 Apr 23;17(5):602. doi: 10.3390/v17050602 (PMC12115982; doi:10.3390/v17050602)
Supplement: Supplementary file 1 [file viruses-17-00602-s001.zip › Table S1.pdf]

Supplemental Table S1. Primers used for quantitative real-time PCR analysis.

Gene	F primer	R primer
NtACS-1	GGATTCGGAGTTTTGTGGGT	CCATTGCTGCTTCTTCTCCATTA
NtACS-5	GGGATACCAGTGGAGC	CAGGAGTGTTGGAGGG
NtACO-1	GGCTCCTTTGCCTGAA	TCCTATGCGCCACTCC
NtACO-5	ACAATGGCGTTAGGGA	TTGCCCACATCCAGAA
NtCTR1	TGGCCTTTGTAGGCATCGAG	CCCTGTCAAGTCCAAATCGAAC
ChiVMV-CP	AAACCCAGCCACAGTCTCGT	ATCTCCGTCCATCATCACCC
NtPOD	GGAGTCAGCAGCCAGCATTT	TTGTTGGGTGGTGAGGTCTT
NtRboh	AACCACAGGGCTACAAATAC	AAGAGCAGAACGAGCATCAC
