# Supplementary material for: Investigation of HCPro-Mediated Ethylene Synthesis Pathway Through RNA-Seq Approaches
Source: Viruses. 2025 Apr 23;17(5):602. doi: 10.3390/v17050602 (PMC12115982; doi:10.3390/v17050602)
Supplement: Supplementary file 1 [file viruses-17-00602-s001.zip › Table S2.pdf]

Supplemental Table S2. Basic summary of RNA-sequencing results.

SampleID	ReadSum	BaseSum	GC(%)	Q20(%)	Q30(%)	Clean reads	Clean bases
HCPro-CK1	*22178023*	*6634134272*	*43.38*	*97.94*	*93.97*	*22,178,023*	*6,634,134,272*
HCPro-CK2	*22166885*	*6639028488*	*43.38*	*98.1*	*94.41*	*22,166,885*	*6,639,028,488*
HCPro-CK3	*20279061*	*6073952586*	*43.38*	*97.74*	*93.58*	*20,279,061*	*6,073,952,586*
HCPro-CK4	*22891059*	*6854712762*	*43.44*	*98*	*94.12*	*22,891,059*	*6,854,712,762*
HCPro-V1	*22319275*	*6683353524*	*42.49*	*98.13*	*94.38*	*22,319,275*	*6,683,353,524*
HCPro-V2	*21131638*	*6326851172*	*42.47*	*98.06*	*94.11*	*21,131,638*	*6,326,851,172*
HCPro-V3	*21581404*	*6460094936*	*42.53*	*98.13*	*94.48*	*21,581,404*	*6,460,094,936*
HCPro-V4	*21980246*	*6522841516*	*42.48*	*98.29*	*94.88*	*21,980,246*	*6,522,841,516*
WT-CK1	*21283422*	*6372784508*	*43.53*	*97.74*	*93.78*	*21,283,422*	*6,372,784,508*
WT-CK2	*20324248*	*6088443406*	*43.51*	*98*	*94.15*	*20,324,248*	*6,088,443,406*
WT-CK3	*20474776*	*6132889838*	*43.55*	*97.72*	*93.69*	*20,474,776*	*6,132,889,838*
WT-CK4	*20529904*	*6147983624*	*43.53*	*97.77*	*93.84*	*20,529,904*	*6,147,983,624*
WT-V1	*20641182*	*6179297710*	*42.5*	*97.76*	*93.74*	*20,641,182*	*6,179,297,710*
WT-V2	*21534835*	*6449153122*	*42.49*	*98.04*	*94.23*	*21,534,835*	*6,449,153,122*
WT-V3	*19986646*	*5979711728*	*42.49*	*98.08*	*94.32*	*19,986,646*	*5,979,711,728*
WT-V4	*21890428*	*6553674874*	*42.48*	*98.02*	*94.17*	*21,890,428*	*6,553,674,874*
